# Are there gender differences in levels of heavy, binge and problem drinking? Evidence from three generations in the west of Scotland

**DOI:** 10.1016/j.puhe.2008.06.001

**Published:** 2009-01

**Authors:** C. Emslie, H. Lewars, G.D. Batty, K. Hunt

**Affiliations:** MRC Social and Public Health Sciences Unit, 4 Lilybank Gardens, Glasgow G12 8RZ, UK

The European region has the highest levels of alcohol consumption in the world. Within Western Europe, the UK has one of the highest levels of binge drinking, and Scotland had the steepest increase in liver cirrhosis mortality rates (an indicator of alcohol harm) during the 1990s.^1^ Hazardous drinking has historically been regarded as a male problem.^2^ However, increases in hazardous drinking among young women have led to suggestions that women are beginning to drink more like men,^3^ and recent media headlines assert that young women in the UK are the ‘worst binge drinkers in the world’. ^4^

There are relatively few studies of drinking patterns in the UK. Existing surveys have tended to sample participants from a narrow age range, and have restricted measures of alcohol consumption which typically focus on average consumption as opposed to drinking patterns. The present study compared the prevalence of three measures of potentially hazardous drinking (‘heavy’, ‘binge’ and ‘problem’ drinking) among men and women using repeat measurement within a longitudinal survey of three age cohorts. The study asked three questions: Are there gender differences in hazardous drinking? If so, are they equally large at two time points (1990 and 2000)? Are they equally large in three cohorts?

Participants were drawn from the West of Scotland Twenty-07 study, a longitudinal study of the health of three age cohorts, in which a detailed questionnaire is administered by trained nurse interviewers approximately every 5 years.^5^ The sociodemographic distribution in the study sample did not differ from a comparable sample of the local population drawn from the UK's 1991 Census samples of anonymized records.

The Twenty-07 study provides detailed information on alcohol consumption. As well as standard scales, it contains a 7-day recall grid which allows the calculation of weekly and daily drinking. Responses were converted to standard units equivalent to 8 g of pure alcohol (half a pint of ordinary beer, lager or cider; a small glass of wine and one measure of spirits each contain 1 unit of alcohol). For context, the proportion of participants who currently drink alcohol and the mean number of units of alcohol consumed in the last week are reported. The main outcomes are three measures of hazardous drinking: heavy drinking (>14 units per week for women, >21 for men^6^), binge drinking (≥7 units in a day in the last week for women and ≥10 for men^6^) and problem drinking in the last year (≥2 on the CAGE questionnaire^7^). Definitions of heavy and binge drinking are contested; the authors have chosen to use definitions widely used in the UK in the period covered by this study. The present measure of binge (or heavy episodic) drinking equates to consuming at least half of the recommended weekly allowance on one occasion.^6^

This paper presents the results from Wave 2 (beginning in 1990) and Wave 4 (beginning in 2000) of data collection. Wave 2 was used as the starting point as this was the first time that the youngest cohort were legally allowed to consume alcohol. In 1990, data were available for 636 men and 705 women in the youngest cohort [born in the early 1970s; mean age at survey 18.6 years, standard deviation (SD) 0.33], 541 men and 673 women in the middle cohort (born in the early 1950s; mean age 40.4 years, SD 0.91), and 576 men and 680 women in the oldest cohort (born in the early 1930s; mean age 59.5 years, SD 0.76). All study members who had data available for either Wave 2 or Wave 4 were included in this study. Response rates for Wave 4 were 63.3%, 78.3% and 78.1% for the youngest, middle and oldest cohorts, respectively.

Men's levels of overall alcohol consumption and hazardous drinking (according to the three indicators) were substantially higher than women's levels in all three cohorts in both 1990 and 2000 (Table 1). Despite the higher cut-offs for men compared with women, substantially higher proportions of men were classified as heavy and binge drinkers; 30.8% vs 11.3% in the youngest cohort, 30.7% vs 7.3% in the middle cohort and 20.8% vs 3.1% in the oldest cohort reported heavy drinking in 1990. Men were more likely than women to be classed as problem drinkers at both time points, with the greatest gender differences in the oldest cohort.

Changes in the ratio of the percentage of male:female drinking suggested that gender differences in heavy drinking reduced over the decade for each cohort; this was because the proportion of men classed as heavy drinkers reduced slightly between 1990 and 2000, while levels for women increased slightly. There was little consistent pattern for binge or problem drinking. For heavy and binge drinking, male:female ratios were smallest in the youngest cohort and largest in the oldest cohort; for example, twice the proportion of men that women reported binge drinking in the youngest cohort, compared 11 times the proportion in the oldest cohort. However, gender differences in problem drinking (an indicator of drinking dependence) were smallest in the middle cohort.

Looking across generations, the youngest cohort had substantially higher levels of binge drinking than other cohorts; in 1990, 47.2% of men in the youngest cohort were classed as binge drinkers compared with 34.6% of men in the middle cohort and 17.2% of men in the oldest cohort. The comparable differences between women were even more striking: 21.7%, 8.8% and 1.5% in the youngest, middle and oldest cohorts, respectively. It was also notable that a higher proportion of respondents in the youngest cohort reported binge drinking rather than heavy drinking at both time points, suggesting that heavy episodic drinking was more common in this cohort than alcohol consumption which was more evenly distributed across the week.

Overall alcohol consumption and levels of hazardous drinking remained much higher among men than women. Rather than women drinking like men,^3^ it is striking that substantial gender differences in hazardous drinking were seen in all three age cohorts in both 1990 and 2000. Indeed, gender differences would be even more marked for heavy and binge drinking if the same definitions were used for men and women, as is the case in some European countries. These data add to the evidence that, in every society where alcohol use has been studied, men drink more than women.^2^

However, these data also indicate that some concern about young women's drinking is justified. Gender differences were generally smallest in the youngest cohort, and recent data from other studies suggest a further narrowing of gender differences in heavy drinking among teenagers.^8^ The present study also found more marked differences across cohorts for women than for men; for example, while binge drinking was very rare among women born in the early 1930s (growing up amid the austerity of war-time Britain and raising children in the 1950s when conventional ideas about women's traditional roles were at their height), it was relatively common among women born in the early 1970s (growing up in an era of legislation around rights to equal pay, anti-sex discrimination in the workplace and paid maternity leave). Thus, one might hypothesize that period effects – such as rapidly changing gender roles and the related changes in attitudes to women's drinking – at least partly explain these more pronounced cohort differences among women. However, an important limitation of these data is that period effects cannot be separated from age or cohort effects.

Another limitation of these data, common to all longitudinal studies, is attrition which, although generally lower in the present cohorts than in other studies, raises concerns about selection bias. In order to investigate whether hazardous drinkers were more likely to drop out of the study, drinking outcomes at Wave 2 were compared for those who responded at Wave 4 with those who did not respond. Statistically significant differences were only found for the oldest cohort. The main analysis was repeated using only the subgroup of participants who responded at both Wave 2 and Wave 4. While the prevalence of binge and problem drinking reduced slightly for men and women in the oldest cohort in 1990, the conclusions were essentially unchanged (results available upon request). This study also has a number of strengths, including its sampling which draws on a general population, very detailed assessment of drinking behaviour and the serial measurement of study participants.

The current advertising campaign to reduce binge drinking in England is aimed at those under 25 years of age.^9^ However, this study found high levels of binge drinking among men in all three cohorts and among women in the youngest cohort. Therefore, these results suggest that public health efforts should concentrate on reducing hazardous drinking among ‘the silent majority of heavy drinkers’, not just on young binge drinkers.^10^

## Figures and Tables

**Table 1:** Prevalence of alcohol consumption for three age cohorts in the West of Scotland Twenty-07 study in 1990 and 2000. Values are percentages (95% confidence intervals)^a^ unless otherwise indicated

Cohort (born)		1990			2000		
		Men	Women	Ratio men: women	Men	Women	Ratio men: women
Youngest (early 1970s)	No. of participants	636	705		381	452	
	Current drinkers	92.0 (89.9, 94.1)	89.8 (87.6, 92.0)	1.0	95.0 (92.8, 97.2)	93.6 (91.6, 96.0)	1.0
	Units (mean)^b^	18.6 (16.8, 20.4)	5.7 (5.0, 6.3)	3.3	17.1 (15.4, 18.9)	6.6 (5.8, 7.4)	2.6

	Heavy drinking^b^	30.8 (27.2, 34.4)	11.3 (9.0, 13.6)	2.7	29.9 (25.3, 34.5)	14.3 (11.1,17.5)	2.1
	Binge drinking^b^	47.2 (43.3, 51.1)	21.7 (18.7, 24.7)	2.2	45.1 (40.1, 50.1)	19.9 (16.2, 23.6)	2.3
	Problem drinkingb,c	—	—	—	17.0 (13.2, 20.8)	4.4 (2.5, 6.3)	3.9

Middle (early 1950s)	No. of participants	541	673		446	532	
	Current drinkers	93.8 (91.8, 95.8)	90.0 (87.7, 92.3)	1.0	93.5 (91.2, 95.8)	90.4 (87.9, 92.9)	1.0
	Units (mean)	17.0 (15.3, 18.6)	5.0 (4.3, 5.6)	3.4	16.8 (15.2, 18.4)	5.8 (5.2, 6.4)	2.9

	Heavy drinking	30.7 (26.8, 34.6)	7.3 (5.3, 9.3)	4.2	28.5 (24.3, 32.7)	10.2 (7.6, 12.8)	2.8
	Binge drinking	34.6 (30.6, 38.6)	8.8 (6.7, 10.9)	3.9	28.9 (24.7, 33.1)	7.0 (4.8, 9.2)	4.1
	Problem drinking	16.0 (12.9, 19.1)	6.7 (4.8, 8.6)	2.4	14.3 (11.1, 17.5)	5.8 (3.8, 7.8)	2.5

Oldest (early 1930s)	No. of participants	576	680		364	462	
	Current drinkers	88.9 (86.3, 91.5)	77.6 (74.5, 80.7)	1.1	87.7 (84.3, 91.1)	74.8 (70.9, 78.7)	1.2
	Units (mean)	13.5 (12.0, 15.0)	2.8 (2.5, 3.2)	4.8	12.4 (10.6, 14.2)	2.8 (2.3, 3.2)	4.4

	Heavy drinking	20.8 (17.5, 24.1)	3.1 (1.8, 4.4)	6.7	18.4 (14.4, 22.4)	3.5 (1.8, 5.2)	5.3
	Binge drinking	17.2 (14.1, 20.3)	1.5 (0.6, 2.4)	11.5	12.1 (8.7, 15.5)	1.1 (0.1, 2.1)	11.0
	Problem drinking	14.9 (12.0, 17.8)	2.6 (1.4, 3.8)	5.7	7.9 (5.1, 10.7)	0.6 (−0.1, 1.3)	13.2

a All gender differences were statistically significant at *P* < 0.001, with the exception of current drinking.

b Heavy drinking, percentage consuming >14 units per week for women, >21 units for men; binge drinking, percentage consuming ≥10 units on a single day in the last week for men, ≥7 for women; problem drinking in the last year, percentage scoring ≥2 out of a possible 4 on the CAGE measure and mean number of units consumed in a week.

c The 1970s cohort were not asked about problem drinking in 1990.
